# Steroid treatment suppresses the CD4^+^ T-cell response to the third dose of mRNA COVID-19 vaccine in systemic autoimmune rheumatic disease patients

**DOI:** 10.1038/s41598-022-25642-z

**Published:** 2022-12-06

**Authors:** Avishai Maliah, Roma Parikh, Oshrat E. Tayer-Shifman, Oded Kimhi, Raz Gepstein, Tami Halperin, Yair Levy, Carmit Levy, Yael Pri-Paz Basson, Shaye Kivity

**Affiliations:** 1 Department of Human Genetics and Biochemistry, Sackler Faculty of Medicine, Tel Aviv University, 69978 Tel Aviv, Israel; 2 Rheumatology Unit, Meir Medical Center, Tchernichovsky St 59, Kfar Saba, Israel; 3 Sackler Faculty of Medicine, Tel-Aviv University, Tel-Aviv, Israel; 4 Department of Internal Medicine A, Meir Medical Center, Kfar Saba, Israel; 5 Department of Ophthalmology, Meir Medical Center, Kfar Saba, Israel; 6 Department of Infectious Diseases, Tel-Aviv Sourasky Medical Center, Sackler Faculty of Medicine, Tel-Aviv University, Tel-Aviv, Israel; 7 Department of Internal Medicine E, Meir Medical Center, Kfar Saba, Israel

**Keywords:** RNA vaccines, Viral infection, Rheumatic diseases, Lymphocyte activation

## Abstract

Prolonged steroid treatment has a suppressive effect on the immune system, however, its effect on the cellular response to mRNA vaccine is unknown. Here we assessed the impact of prolonged steroid treatment on the T-cell and humoral response to the SARS-CoV-2 spike (S) peptide following the third dose of the BNT162b2 vaccine in systemic autoimmune rheumatic disease patients. We found that CD4 T-cell response to the S peptide in patients on high-dose long-term steroid treatment showed significantly less S-peptide specific response, compare to low-dose or untreated patients. Remarkably, these results were not reflected in their humoral response, since almost all patients in the cohort had sufficient antibody levels. Moreover, S-peptide activation failed to induce significant mRNA levels of IFNγ and TNFα in patients receiving high-dose steroids. RNA-sequencing datasets analysis implies that steroid treatments' inhibitory effect of nuclear factor kappa-B signaling may interfere with the activation of S-specific CD4 T-cells. This reveals that high-dose steroid treatment inhibits T-cell response to the mRNA vaccine, despite having sufficient antibody levels. Since T-cell immunity is a crucial factor in the immune response to viruses, our findings highlight the need for enhancing the efficiency of vaccines in immune-suppressive patients, by modulation of the T-cell response.

## Introduction

Systemic autoimmune rheumatic diseases (SARDs) encompass a group of rare inflammatory conditions associated with autoimmune dysregulation, wherein glucocorticoids and immunosuppressive medication are the treatment of choice, rendering these populations with a suppressed immune system^1^. High-dose steroid treatment, but not Disease-modifying anti-rheumatic drugs (DMARDs) and anti-TNFα treatment, is significantly associated with higher odds of hospitalization with severe COVID-19 infection^2^. A better understanding of the ability of immunosuppressive therapies in influencing the response to the vaccine may contribute to reducing the risk of SARDS patients during the COVID-19 pandemic.

BNT162b1 mRNA vaccine can protect against COVID-19 severe disease through multiple beneficial mechanisms, including increased antibody titers and induction of S-specific CD4^+^ and CD8^+^ T-cell^3^. Previous studies have shown that the BNT162b2 vaccine is immunogenic in SARDs patients^4^ and that vaccinated patients have better outcomes than their unvaccinated counterparts^5^. However, the possibility that the immunosuppressive drugs used to treat SARDs might impair patients’ responses to mRNA vaccines is still a major concern.

Researchers suggest an important role of CD4^+^ and CD8^+^ T-cells responses in the clearance of SARS-CoV-2 infection^3,6^. In particular, CD4^+^ T-cell responses to the vaccine are crucial in creating antiviral immunity and CD4^+^ T-cells provide protection against the lethality of SARS-CoV-2 even in the absence of CD8^+^ T-cells^7^. For this reason, some COVID-19 vaccination efforts are focused on elicitation of CD4^+^ or CD8^+^ T-cells^3^.

In this study, we assessed the serological and cellular immune responses to the third dose of the BNT162b2 vaccine in SARDs patients and found that prolonged high-dose steroids treatment has an inhibitory role on S-specific CD4 T-cells response, despite the vaccine’s success in inducing high antibody titers. Assessment of gene expression of T-cells from patients treated with high-dose steroids, re-analysis of the published RNAseq. datasets on the effect of steroids on CD4 cells and about gene expression of virus-specific CD4 cells indicate that steroids specifically inhibit Th1 cytokines TNFα and interferon-γ, further showing inhibition of nuclear factor kappa-B signaling by steroids in CD4 T-cells in the response to the mRNA vaccine.

## Results

### Participant characteristics

Our final cohort consisted of 29 SARDs patients who had received three doses of the BNT162b2 mRNA vaccine and seven vaccinated HCs (five with three doses; two with two doses) (Table 1). Five HCs (three with three doses; two with two doses) and 19 SARDs patients were evaluated for antibody titers, whereas, seven HCs and 19 SARDs patients were evaluated for cellular response using intracellular staining flow-cytometry. Additionally, 10 SARDs patients participated in the validation experiment (qRT-PCR). The average age of the group was 55 years old, and females accounted for 71% of the cohort. The mean time of vaccination from the last dose was 87.6 days. Rheumatoid arthritis was the most common disease (n = 8) followed by systemic lupus erythematosus (n = 5), vasculitis (polyarteritis nodosa and giant cell arteritis) (n = 4), sarcoidosis (n = 4), systemic sclerosis (n = 4) and uveitis, polymyalgia rheumatic, familial mediterranean fever, and sjogren syndrome (n = 1 for each). Nineteen out of the 29 SARDs patients were treated with immunomodulatory medications, of which glucocorticoids (n = 16) were the most common, prednisone as monotherapy (n = 4), prednisone in combination with other immunomodulatory medications (n = 12), methotrexate (n = 5), and mycophenolate mofetil (n = 3).

**Table 1:** Patient demographics and clinical data.

Group	Gender	Age	Diagnosis	Treatment	Gluco corticoids (mg/day)	Steroid group bifurcation for analysis	Time from vacc. (days)	Vacc dose
**Flow cytometry and Humoral response experiment**								
HC	F	66		None		Untreated	88	3
HC	F	50		None		Untreated	73	3
HC	F	73		None		Untreated	59	3
HC	M	50		None		Untreated	50	3
HC	F	43		None		Untreated	43	3
HC	F	26		None		Untreated	239	2
HC	F	52		None		Untreated	206	2
Un SARD	F	22	SLE	HQ		Untreated	82	3
Un SARD	F	22	FMF	None		Untreated	53	3
Un SARD	F	66	SS	None		Untreated	79	3
Un SARD	F	41	SLE	HQ		Untreated	85	3
Un SARD	F	44	SSCL	Iloprost, bosentan		Untreated	73	3
T SARD	F	68	GCA	None	≤ 10	Non/low str	82	3
T SARD	M	65	RA	Salazopyrin, infliximab	10	Non/low str	74	3
T SARD	F	65	PAN	Methotrexate	> 20	High str	84	3
T SARD	F	48	RA	Methotrexate		Non/low str	45	3
T SARD	F	47	SLE	HQ, Belimumab	< 10	Non/low str	42	3
T SARD	F	65	RA	leflunomide, mabthera		Non/low str	86	3
T SARD	M	63	SARC	Infliximan	> 10	High str	94	3
T SARD	F	52	SARC	Imuran	> 20	High str	48	3
T SARD	M	70	RA	leflunomide, mabthera		Non/low str	96	3
T SARD	M	51	UV	Cellcept	< 5	Non/low str	93	3
T SARD	F	57	SARC	Methotrexate	> 10	High str	55	3
T SARD	F	26	SLE	HQ, cellcept	> 10	High str	43	3
T SARD	M	88	GCA	None	> 20	High str	63	3
T SARD	F	65	SLE	HQ, cellcept	> 10	High str	104	3
**qRT-PCR experiment**								
Un SARD	F	57	SSCL	None		Control	115	3
Un SARD	F	52	SSCL	none		Control	121	3
Un SARD	F	72	PMR	None		Control	132	3
Un SARD	M	85	RA	None		Control	140	3
Un SARD	M	66	DM	None		Control	135	3
T SARD	M	86	SARC	None	10–20	High-steroid	124	3
T SARD	F	45	RA	HQ, MTX	10–20	High-steroid	50	3
T SARD	F	36	RA	MTX, golimumab	10–15	High-steroid	47	3
T SARD	F	71	PAN	Cytoxan	10–20	High-steroid	120	3
T SARD	M	58	RA	None	> 20	High-steroid	115	3

HC, healthy control; UN SARD, untreated SARD; T SARD, treated SARD; SARD, systemic autoimmune rheumatic disease; Vacc., vaccine; Exp., experiment; SLE, systemic lupus erythematosus; FMF, familial mediterranean fever; SS, Sjogren's Syndrome; SSCL, systemic sclerosis ; GCA, giant cell arteritis; RA, rheumatoid arthritis; PAN, polyarteritis nodosa; SARC, sarcoidosis; PMR, polymyalgia rheumatic; UV, uveitis; HQ, hydroxychloroquine; DM, dermatomyositis F, female; M, male.

### Humoral and cellular response to the BNT162b2 vaccine in SARDs immunosuppressed patients

**Figure 1:**
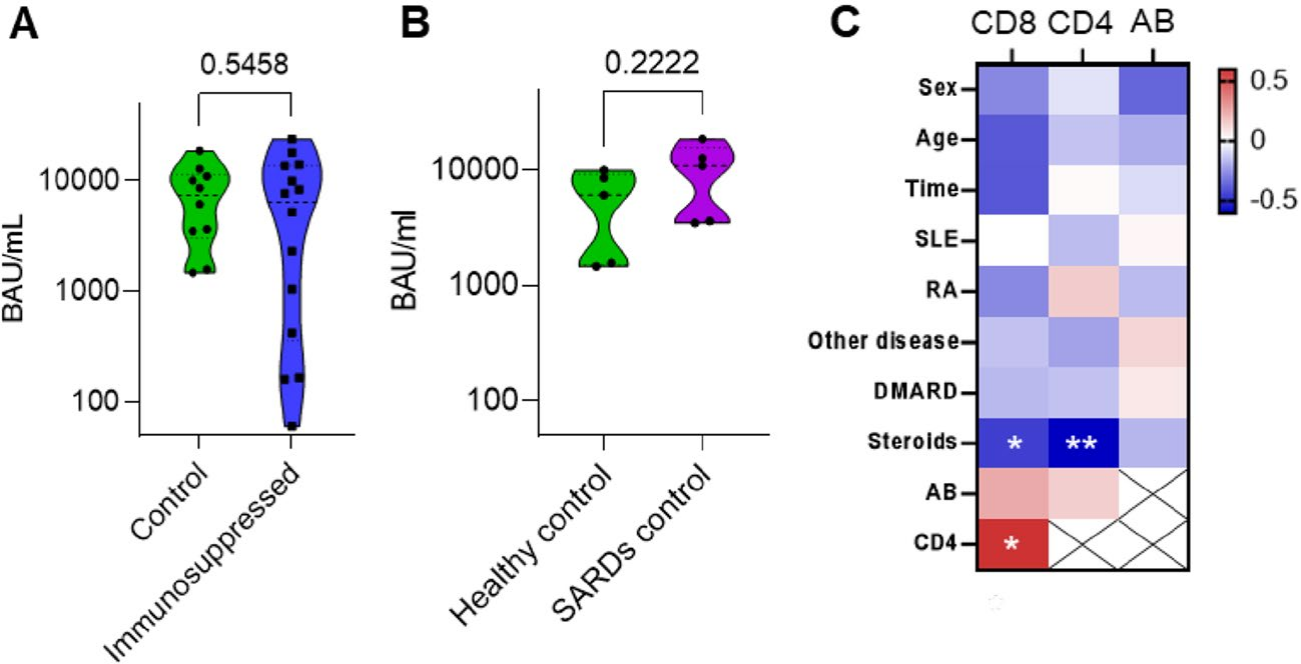
Humoral and T-cell response to the BNT162b2 vaccine in SARDs immunosuppressed patients. (**A**) S-specific antibody level (AU/ml) of HCs and SARDs patients who were not treated with immunosuppressant’s (green, control) (n = 10) versus SARDs patients who were treated with any immunosuppressant’s (blue, immunosuppressed) (n = 14). The two-tailed Mann–Whitney test P value is indicated. (**B**) Comparison of S-specific antibody level (BAU/ml) between HCs (green) (n = 5) and SARDs patients who were not treated with any immunosuppressive drug (violet, SARDs control) (n = 5). The two-tailed Mann–Whitney test P value is shown in the graph. (**C**) Heatmap of Spearman R correlation between patients’ demographic and clinical parameters with S-specific antibody (AB) level (BAU/ml) and the percentage of S-specific CD4 and CD8 cells. The parameters examined were sex (0-female, 1-male), age (years), time (days post-vaccination), diagnosis of RA (0-no, 1-yes), diagnosis of SLE (0-no, 1-yes), diagnosis of other SARDs (0-no, 1-yes), treatment with DMARDs (0-no, 1-yes), treatment with steroids (0-no, 1-low dose, 2- high dose), and treatment with other immunosuppressive drugs (0-no, 1-yes). The correlation between AB level and CD4 and CD8 level is also represented. Significant correlations are marked with * for *P* < 0.05 and ** for *P* < 0.01.

Antibody titer measurements were analyzed for a total of 24 participants including: 10 control participants (five HCs, five SARDs patients without immunomodulatory treatment) and 14 immunosuppressed SARDs patients. All 24 participants had antibody levels higher than the minimal threshold (> 50AU/mL = 7.1 BAU/mL; Fig. 1A,B). No significant differences were found in the antibody levels among the HCs compared to untreated SARDs patients (SARDs control) (Fig. 1B). Further, no significant differences were found between the control group (HCs and SARDs patients without immunomodulatory treatment) and immunosuppressed SARDs patients (Fig. 1A) despite the relatively higher age of the immunosuppressed patients (Table S1). We observed no significant differences in the gender and time of vaccination among the HCs, untreated SARDs, and treated SARDs patients (Table S1). Additionally, we did not observe significant correlations in the antibody levels and age, gender, time from vaccination, type of rheumatic diseases, or treatment group (Fig. 1C).

**Figure 2:**
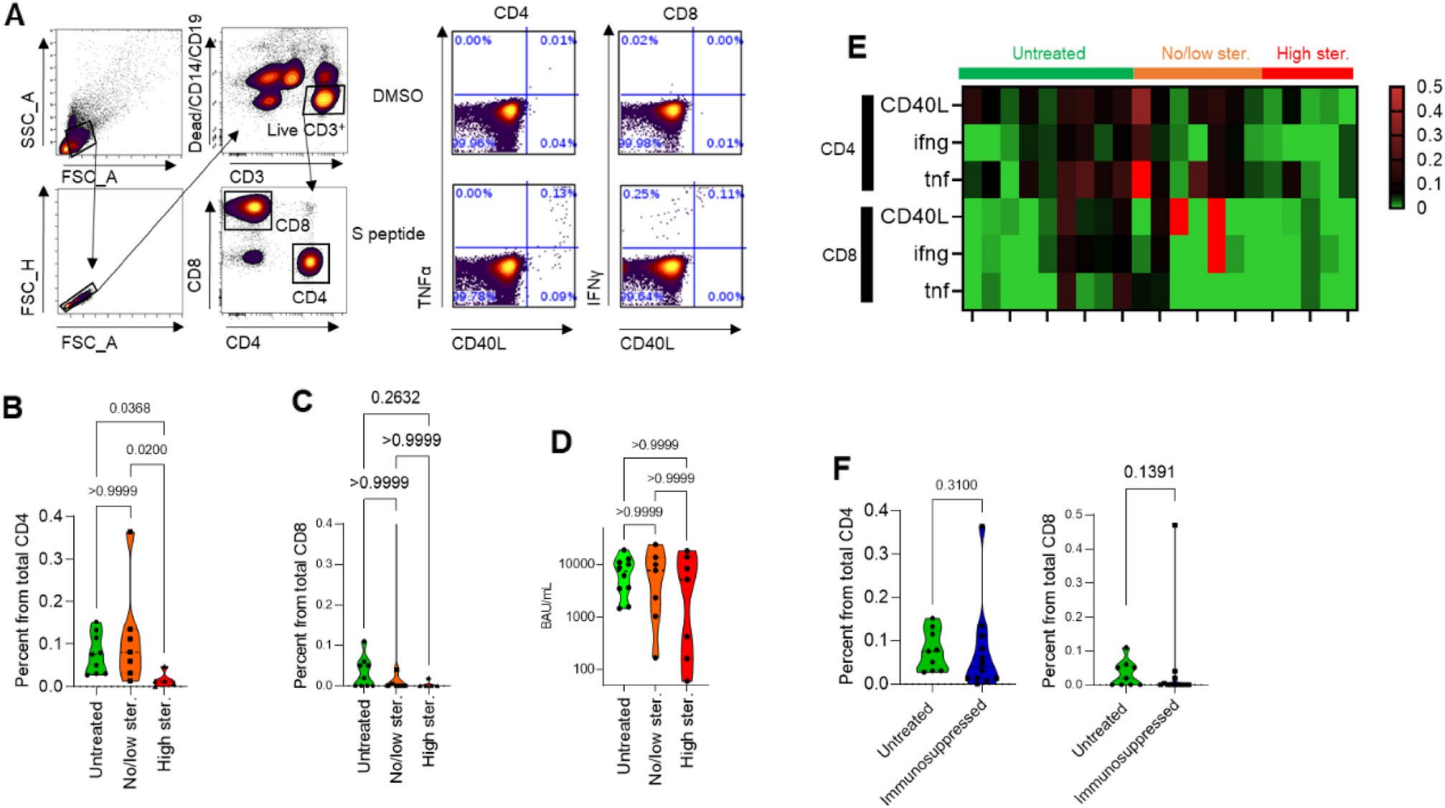
Impaired cytokine response to S peptide in BNT162b2 vaccinated SARDs patients treated with a high-dose of steroids. (**A**) Gating strategy of the flow cytometry analysis. CD4 and CD8 are defined as CD3^+^, CD19^−^, CD14^−^, and dead cell marker negative. Representative image of a patient’s S-specific CD4 (left panel) and CD8 (right panel). The upper part represents the DMSO negative control, and the lower part represents activation with the S peptide. CD4 (**B**) and CD8 cells (**C**) between participants who were not treated with any immunosuppressive drug (green, untreated group), those who were treated with non- or low-dose immunosuppressant’s (orange, non/low str.), and participants who were treated with a high-dose of steroids (red, high str.). The percentages displayed within the quarters are from all CD4 or CD8 cells. Two-tailed Kruskal–Wallis test with Dunn correction *P* value are indicated. (**D**) Comparison of S-specific antibody level (BAU/ml) among the participants who were not treated with any immunosuppressive drug (untreated group), participants who were treated with non- or low-dose steroids (non/low str. group), and participants who were treated with a high-dose of steroids (high str. group). Two-tailed Kruskal–Wallis test with Dunn correction P values is marked in the graph. (**E**) Heatmap of HCs, non- or low-dose steroids (Non/low str.) and high-dose steroid (High str.) groups with the expression of activation markers on participants’ T-cells. The colors represent the percentage of CD4 or CD8 cells that were positive to at least one of the activation markers from total CD4 or CD8 cells, respectively. Each column represents a participant, and each row represents a cell type with the expression of activation markers. (**F**) Percentage of S-specific CD4 (left panel) and CD8 (right panel) of HCs and SARDs patients who were not treated with an immunosuppressant (green, non-treated) versus SARDs patients who were treated with any immunosuppressive drug (blue, immunosuppressed). Two-tailed Mann–Whitney test *P* value are indicated.

In HCs, exposure of T-cells to S peptide-induced CD40L^+^ TNFα^+^ CD4 cells and CD4OL^+^ IFN_γ_^+^ CD8 cells, whereas no change was observed in unstimulated cells (Fig. 2A). Hence, CD40L^+^ TNFα^+^ CD4 and CD4OL^+^ IFN_γ_^+^ CD8 cells were defined as S-specific T-cells. Correlation analysis showed a negative correlation between the steroid treatment and S peptide-specific CD4 and CD8 levels (P = 0.004 and 0.034 respectively), whereas there were no significant correlations between immunosuppressive or other treatments to low levels of S-specific T-cells (Fig. 1C). Additionally, we found a significant positive correlation between S peptide-specific CD4 and CD8 levels (*P* = 0.008, Fig. 1C).

Segregation of participants based on steroid treatment, revealed that high-dose steroid group (> 10 mg prednisone/day) had significantly fewer S-specific CD40L^+^ TNFα^+^ CD4 cells (0.01%) compared to untreated patients (median = 0.075%; *P* = 0.036) or patients treated with low-dose steroids (median = 0.081%; *P* = 0.02; Fig. 2B). In the high-dose steroid group, almost no S peptide-specific CD40L^+^ IFN_γ_^+^ CD8 cells (median = 0%) were found. However, the difference between the groups was not significant (median: no steroids = 0.03% low = 0; *P* = 0.19; Fig. 2C). We found no significant differences in antibodies level (Fig. 2D), or age, gender, and time from vaccination between HCs and the high- or low-dose steroid groups (Table S1). The heatmap shows the level of S peptide-specific cells for each marker individually (CD40L^+^ CD4 or CD8; IFN_γ_^+^ CD4 or CD8; and TNFα^+^ CD4 or CD8) demonstrating the difference in the T-cell response between the low-, high-dose steroids and the untreated groups, that was found for all the markers we examined (Fig. 2E).

Moreover, a comparison of the non-treated group (HCs and rheumatic patients—no immunosuppressant’s) with the immunosuppressant treated group revealed no significant differences in S peptide-specific CD4 or CD8 cells (Fig. 2F). These findings imply that in our patient cohort, only high-dose steroid treatment is associated with less S-specific CD4 cells.

### Specific inhibitory effects of steroids on T-cells response to SARS-CoV-2

**Figure 3:**
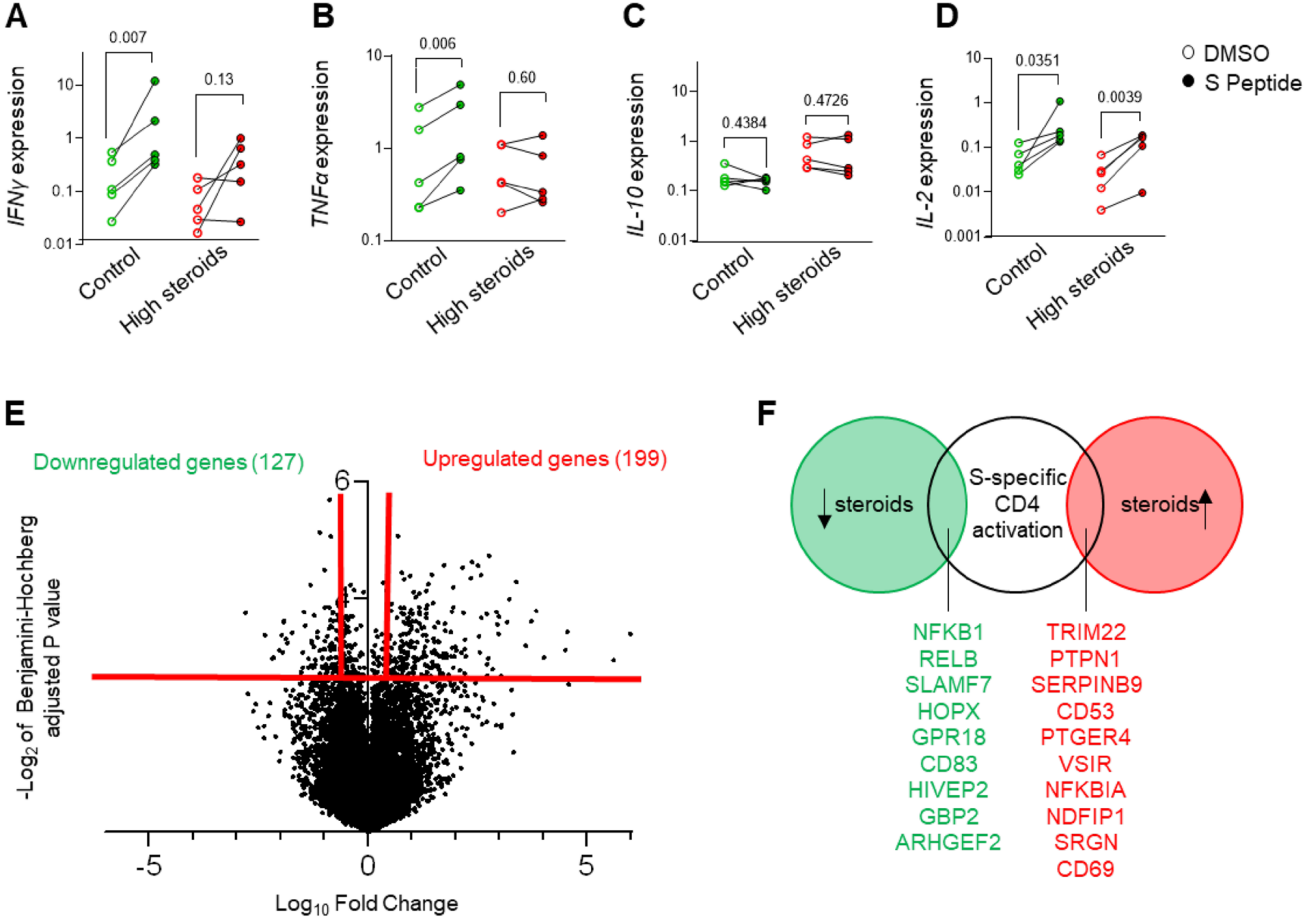
Specific inhibitory effects of steroids on T-cells response to SARS-CoV-2. mRNA expression of *IFNγ* (**A**), *TNFα* (**B**), IL-10 (**C**), and IL-2 (**D**); relative to housekeeping gene expression (*HPRT*), in SARDs patients who were not treated with steroids (green, control) versus SARDs patients who were treated with high-dose of steroids (red, high steroids). For each patient, an empty circle indicates the level of expression in the DMSO negative control and a filled circle indicates the expression after 24 h of activation with S peptide. Paired t test P value are provided. (**E**) Volcano plot depicting gene expression upon steroid treatment in vitro on CD4 T-cells. Red lines indicate the threshold for determining the differentially expressed genes. (**F**) Venn diagram showing the overlap between genes that were differentially expressed upon steroid treatments in vitro on CD4 T-cells and genes upregulated on S-specific activation of CD4 cells. The overlap with the upregulated genes is represented in Red and the downregulated genes are represented in green.

To determine the effect of steroid treatment on T-cells response to the BNT162b2 vaccine, we analyzed mRNA expression of cytokines from SARDs patients with three doses of the BNT162b2 vaccine: 5 non-treated SARDs patients (control) and 5 SARDs patients treated with steroids (high-steroids, ≥ 10 mg of prednisone), in 3 out of those patients the prednisone was combined with other immunomodulatory medications. In the control group, the comparison between the S-peptide stimulation vs DMSO stimulation revealed a significant increase in the mRNA expression of *IFNγ* (*P* = 0.007; Fig. 3A) and *TNFα* (*P* = 0.006; Fig. 3B) in the S-peptide stimulated group. No such differences were observed between the S-peptide stimulation vs DMSO stimulation in the patients receiving high-dose steroid treatment (*P* = 0.13 and *P* = 0.60; Fig. 3A,B respectively). Despite the ability of the steroids to induce human T-cell populations to secrete IL-10^8^, we did not see any significant changes in *IL-10* mRNA levels upon S peptide activation (Fig. 3C). On the other hand, *IL-2* mRNA levels were significantly increased in the S peptide-stimulated group of all patients (Fig. 3D). These results imply that patients receiving high-dose of steroids might have a partial T-cells response to S-peptide, however, their ability to secrete Th1 cytokines such as *TNFα* and *IFNγ* is impaired.

The response of the immune cells to glucocorticoids is highly cell-type dependent^9^. In order to specifically investigate the ability of steroids to inhibit virus-specific CD4 cells, we performed analysis on the two published RNAseq. datasets: (A) that examined the effect of in vitro steroid therapy on gene expression after 6 hours^9^, and (B) RNAseq. dataset showing genes that were changed in virus-specific CD4 cells upon activation with the S peptide^10^. We found that 199 genes were upregulated (log10 fold change > 0.1, FDR < 0.1), and 127 genes were downregulated upon steroid treatment (Fig. 3E). Enrichment analysis of the transcription factors that regulate these genes showed that the genes that were decreased upon steroids treatment were majorly regulated by NF-κB signaling (Table S4A-B). To examine specifically the ability of steroids to inhibit virus-specific CD4 cells, we performed overlap between differentially expressed genes upon steroids to the genes that were increased upon activation of virus-specific CD4 cells (Fig. 3F), revealing 6 genes associated with NF-κB signaling (NFKB1, RELB, CD83^11,12^ SLAMF7^13^, HIVPV2^14^, ARHGEF2^15^). On the other hand, we also found the NFKBIA (NF-κB inhibitor A) gene, which is a negative regulator of the NF-kB signaling. NFKBIA is a well-known gene that is induced upon steroid treatment as well as induced upon activation of specific CD4 cells. GBP2 and CD83 are downregulated upon steroids treatment and are known as interferon-inducible genes^12,16^. Further, we found that NF-κB signaling was among the top transcription factors that regulate the genes that were upregulated upon CD4 T-cell S peptide stimulation (Table S4C). Taken together our data demonstrate that steroids' impairment of cytokine secretion of the virus-specific CD4 cells is caused by inhibition of the NF-κB signaling.

## Discussion

Our data suggest that most of the SARDs patients that were vaccinated with three doses of the BNT162b2 vaccine had a sufficient humoral and cellular response to the vaccine. However, long-term treatment with high-dose steroids had an inhibitory effect on T-cell immune responses to the BNT162b2 vaccine which might partly be attributed to the abrogation of the NF-κB signaling upon steroid treatment. Moreover, in our patient cohort, only high-dose steroid treatment was associated with less S-specific CD4 cells.

Several studies have shown that SARDs patients have a higher chance for COVID-19-related complications, including death, specifically when treated with immunosuppressant’s such as anti-B cell therapy and glucocorticoids (> 10 mg/day)^2^. Our study may explain why SARDs patients treated with glucocorticoids have a higher risk for severe COVID-19 despite having high antibody titers.

The inhibitory effect of steroids on the immune system was reported previously^17^, however, our study is the first to examine the inhibitory effect of steroids in the context of T-cell response to mRNA vaccines. The mRNA vaccines are effective at inducing T helper 1 (Th1) responses, which enhance cytotoxic responses to intracellular pathogens^18,19^. Th1 cells were found to be more sensitive to glucocorticoid-induced apoptosis and cytokine suppression compared to Th2 and Th17 cells^20^. Our finding demonstrates that steroid treatment inhibits Th1 cytokines *IFNγ* and *TNFα* expression implying that steroids inhibition of virus-specific CD4 cells is related to the impairment of the Th1 response. Further, our bioinformatics analysis suggest that the most significant effect of steroids was related to NF-κB signaling inhibition nevertheless also pointing towards other pathways that can contribute to steroid inhibition of CD4 T-cells. While the ability of steroids to inhibit NF-κB signaling is well established^17^, the role of this pathway in response to mRNA vaccine is still less known.

Previous studies have demonstrated that steroids increase the survival, maturation, and differentiation of regulatory T-cells^21^. Here, we did not see an upregulation in IL-10 expression levels in T- cells upon steroid treatment. More studies focusing on the effect of steroids on regulatory cytokine expression and T-cells in the context of mRNA vaccines might contribute to a better understanding of the role of steroids in inhibiting the cellular response to mRNA vaccine. Further, studies show that anti-B cell therapy and glucocorticoid treatment were found to reduce the humoral response to two doses of the BNT162b2 vaccine^4,22^. However, the third dose of the vaccine was shown to increase the antibody titers also in immunosuppressed patients^23,24^. The possible explanation to our results about not finding significant difference in the level of antibodies between SARD patients who are treated with steroids and those who are not treated with steroids is that the third dose of the vaccine succeeds in raising the level of antibodies, even in steroids treated patients, in a more optimal way than the cellular response.

Due to our small study population and the diverse types of diseases and treatments, our ability to draw conclusions about treatments other than high-dose steroids is limited. Further, despite no differences found in the age, gender, and time from vaccination between the high steroid groups compared to other groups, we were not able to eliminate that other factors including differences in the treatments or other clinical factors such as the type of disease may influence our results. Future studies exploring the effect of different immunomodulatory treatments and the underlying molecular mechanism responsible for the effect of immunosuppressive therapies on T-cells in the context of mRNA vaccines are warranted.

## Materials and methods

### Study population

Human cohort consisting of HCs with no underlying diseases and patients with SARDs (age 22–73 years) were recruited from the rheumatology clinic at Meir Hospital (Kfar Saba, Israel) between October and December 2021 in this retrospective study. Five HCs had received three doses of the mRNA-based BNT162b2 vaccine and two HCs received two doses. All patients in the study had received three doses. The exclusion criteria for the cohort included, patients with a previously positive PCR COVID-19 test or who were clinically diagnosed with COVID-19, pregnancy, infections, malignancies and age ≤ 18 or ≥ 80 years old. All SARDs patients had a stable disease and were treated on ambulatory basis. The sampling technique used for the study was convenience sampling. Blood was drawn from the participants after a minimum of 4 weeks post-vaccination. All participants provided written informed consent to participate in the study. This study was approved by the Institutional Review Board of the Meir Medical Center and is registered under identifier 0056–21 and all experiments were performed in accordance with relevant guidelines and regulations. Patients’ data are summarized in Table 1 and Table S1.

### Serum preparation

Whole blood was collected in a serum separator tube (BD Vacutainer, #365328, BD, Franklin Lakes, NJ, USA) from each participant. The sample was allowed to clot for 30–60 min at room temperature (RT), followed by centrifugation at 2000 RCF for 10 min at 4 °C. The serum was collected into an Eppendorf tube and frozen at − 80 °C until further use.

### Humoral immunity induced by the vaccine

The presence of anti-SARS-CoV-2 IgG antibodies in the serum was evaluated using the SARS-CoV-2 IgG chemiluminescent microparticle immunoassay (CMIA) (Abbott, Sligo, Ireland). This test targets the RBD (receptor binding domain) located on the S1 subunit of the spike protein. Results were provided in arbitrary units (AU) per milliliter, as defined by the manufacturer, ranging from 0 to 40,000 AU/mL for anti-S1/RBD antibodies. A level of > 50 AU/mL (7.1 BAU/ml) was considered to be positive.

### Stimulation of PBMCs with SARS-CoV-2 peptide pools

PBMCs were isolated from whole blood using Ficoll-Paque Plus gradient density (GE Healthcare, Chicago, IL, USA). PBMCs were seeded into 96-well cell culture plates (1*10^6^ cells/well) in RPMI media supplemented with 5% Human AB-serum and stimulated with SARS-CoV-2 PepTivator peptide pools consisting of 15-mer sequences with 11 amino acid (aa) overlap covering the complete protein coding sequence (aa 5–1273) of the surface or S glycoprotein (GenBank MN908947.3, Protein QHD43416.1) according to the manufacturer’s instructions (Miltenyi Biotech, #130-128-156; Westphalia, Germany). Briefly, the PepTivator was diluted in sterile water/10% DMSO solution and used at a concentration of 1 µg/ml. As the positive control, cells were stimulated with CytoStim; as the negative control, cells were treated with 10% DMSO solution and incubated for 2 h at 37 °C, 5% CO_2_. This was followed by a 4 h incubation with Brefeldin A at 37 °C, 5% CO_2_ to inhibit the transport of proteins to the cell membrane.

### Flow cytometry

Upon stimulation with the SARS-CoV-2 PepTivator, PBMCs were stained using fluorophore-conjugated antibodies. A master mix containing all the antibodies was prepared directly prior to staining. At first, the cells were stained using live/dead marker Viobility 405/452 dye (Miltenyi Biotech) for 10 min at RT in the dark. Following fixation and permeabilization, the cells were stained using fluorochrome-conjugated antibodies (Table S2) for 10 min at RT in the dark. All samples were washed thoroughly with PBS containing 0.5% bovine serum albumin (AMERESCO, USA) and 2 mM EDTA. Data were acquired using the Cytoflex 5L cytometer (Beckman Coulter) followed by analysis using Cytobank software (Beckman Coulter, Brea, CA, USA). Live CD14^–^, CD20^–^, CD3^+^, CD4, and CD8 T-cells specific to SARS-CoV-2 S protein were defined based on the double expression of two activation markers exhibiting the highest expression levels of CD40L^+^ TNFα^+^ CD4 T-cells and CD40L^+^ IFNγ^+^ CD8 T-cells. The percentage of S-specific peptide CD4 for each patient was calculated as the percentage of CD40L^+^ TNFα^+^ CD4 in the S peptide-stimulated sample minus the percentage of CD40L^+^ TNFα^+^ CD4 in the DMSO control sample. The same calculation was performed for S-specific peptide CD8 and for each marker (CD40L, TNFα, and IFNγ) separately.

### T-cell enrichment, RNA purification, and qRT-PCR

Ten out of 29 SARDs patients were recruited for this experiment. Five of these patients received prednisone ≥ 10 mg/day (high-dose steroid group), three patients received prednisone as monotherapy and two patients received prednisone in combination with other immunosuppressive medications. The other five patients were not being treated with steroids or other immunosuppressants (control group). Blood samples were collected, and T-cells were enriched from the PBMCs based on the negative selection technique using the Pan T-cell Isolation Kit following the manufacturer’s instructions (Miltenyi Biotech, 130-096-535).

Total RNA was extracted from the T-cells after 24 h of stimulation with the S peptide or DMSO using TriZol (Invitrogen, Waltham, MA, USA) according to the manufacturer’s instructions. RNA was quantified by OD260 nm/OD280 nm measurement (NanoDrop, ThermoFisher). For mRNA analysis, cDNA was prepared using the qScript cDNA synthesis kit (Quantabio, Beverly, MA, USA) and subjected to qRT-PCR using the PerfeCTa SYBR green FastMix (Quantabio). Analysis of *IFNγ*, *TNF*α, IL-2, and IL-10 was performed by considering the logarithmic values for the arbitrary values of the DMSO control and S peptide-stimulated samples. The oligonucleotide sequences used are listed in Table S3.

### RNA seq. data analysis

Data analysis showing the effect of in vitro steroid treatment on CD4 T-cells was conducted using RNAseq. From Franco et al. article^9^. The row read counts were first normalized to transcript per million (TPM), followed by an examination of the differentially expressed genes 6 h after the steroid was taken (22.7 µM of methylprednisolone). Genes with < 50 reads in any samples were filtered out. The significance was determined using paired two-tailed TTest (for ratio) and FDR correction with FDR < 0.1 and log fold change of > 0.5 or < − 0.5 defined as a gene with significant change upon steroid treatment. The overlap was performed between genes from this dataset and genes that increase in virus-specific CD4 cells upon activation taken from the single-cell RNAseq analysis of Fischer et al.^10^. The adjusted P value significance level was set at less than 0.05. We conducted an over-representation analysis using WebGestaly (WEB-based Gene SeT AnaLysis Toolkit) to identify transcription factor(s) that regulate the differentially expressed genes upon steroid treatment and the genes that increase upon activation of virus-specific CD4 cells. The top ten transcription factor targets of these genes are shown in Table S4A-C.

### Statistical analysis

We used the two-tailed Mann–Whitney U test for comparisons between two groups and the two-tailed Kruskal–Wallis test with Dunn correction for comparisons among three groups. Spearman correlation was used for correlation analysis between a patient’s clinical and demographic parameters and the antibodies and S-specific T-cell levels. A paired two-tailed t test ratio was used to compare the IFNγ, TNFα, IL-2, and IL-10 mRNA expression levels between the DMSO control and S peptide-stimulated samples. *P* values < 0.05 were considered to be statistically significant. The statistical analysis and graphing were performed using GraphPad Prism 9 software.

## Supplementary Information

Supplementary Legends.

Supplementary Tables.

## Data Availability

All the data relevant to this study are included in the manuscript, tables, and figures. Additional data will be provided upon request.
