# Supplementary material for: Steroid treatment suppresses the CD4+ T-cell response to the third dose of mRNA COVID-19 vaccine in systemic autoimmune rheumatic disease patients
Source: Sci Rep. 2022 Dec 6;12:21056. doi: 10.1038/s41598-022-25642-z (PMC9727118; doi:10.1038/s41598-022-25642-z)
Supplement: Supplementary file 1 — Supplementary Legends. [file 41598_2022_25642_MOESM1_ESM.docx]

**Supplementary Table 1.** No significant difference in sex, age, or time from vaccination between the groups was found in the main experiment. Comparisons were conducted between treated and untreated groups (upper panel) or among the untreated group, patients who were treated without or with low-dose steroids (Low/no ster.), and those who were treated with high-dose steroids (high ster.; lower panel). The median age and time from vaccination with 95% confidence intervals are indicated. The chi-square *P* value of the sex comparisons are listed, and the Mann-Whitney *P* value and Kruskal-Wallis *P* value are indicated for the age and time from vaccination comparisons in the upper and lower panels, respectively.
