# Supplementary material for: Steroid treatment suppresses the CD4+ T-cell response to the third dose of mRNA COVID-19 vaccine in systemic autoimmune rheumatic disease patients
Source: Sci Rep. 2022 Dec 6;12:21056. doi: 10.1038/s41598-022-25642-z (PMC9727118; doi:10.1038/s41598-022-25642-z)
Supplement: Supplementary file 2 — Supplementary Tables. [file 41598_2022_25642_MOESM2_ESM.pdf]

Supplementary Table 1

	non treated (n=12)	treated (n=14)	P value
gender (is female)	91.67%	64.29%	0.0985
Mean age (STD)	46.25 (17.02)	59.29 (14.34)	0.044
Median time from vac. (IQR)	76 (54.5-87.25)	78 (47.25-93.25)	0.8496

	non treated (n=12)	non or low steroids (n=7)	high steroids (n=7)	P value
gender (is female)	91.67%	57.14%	71.42%	0.2089
Mean age (STD)	46.25 (17.02)	59.14 (10.02)	59.43 (18.57)	0.1386
Median time from vac. (IQR)	76 (54.5-87.25)	82 (45-93)	63 (48-94)	0.9415

Supplementary Table 1. Comparison of sex, age, and time from vaccination between the groups were assessed in the main experiment. Comparisons were conducted between treated and non-treated groups (upper panel) or among the untreated group, patients who were treated without or with a low dose of steroids (Low/non str.), and those who were treated with a high dose of steroids (High str.; lower panel). The chi-square P value of the sex comparison are listed, Mann-Whitney P value and Kruskal-Wallis P value are indicated for the time from vaccination comparisons , and Student t test and Anova test for age comparisons in upper and lower panel respectively.

Supplementary Table 2. Antibodies used in the flow cytometry analysis

Marker	Fluorophore	Clone
CD3	APC	REA613
CD4	Vio®-Bright B515	REA623
CD8	VioGreen™	REA734
CD20	VioBlue®	REA780
CD14	VioBlue®	REA599
IFNγ	PE	REA600
TNFα-	PE-Vio®770	REA656
CD154	APCVio®770	REA238

Supplementary Table 3. Human oligonucleotide sequences

IFNγ	sense	5’-GCATCGTTTTGGGTTCTCTTG-3’
	anti-sense	5’-AGTTCCATTATCCGCTACATCTG-3’
TNFα	sense	5’-CAAGCCTGTAGCCCATGTT-3’
	anti-sense	5’-TCAGCTCCACGCCATTG-3’
IL-2	sense	5’-TTTACATGCCCAAGAAGGC-3’
	anti-sense	5’-CACTTCCTCCAGAGGTTTGA-3’
IL10	sense	5’-TCAAGGCGCATGTGAACT-3’
	anti-sense	5’-ACGGCCTTGCTCTTGTTT-3’
HPRT	sense	5’-ATGGACAGGACTGAACGTCTTG-3’
	anti-sense	5’-GGCTACAATGTGATGGCCTC-3’.

Supplementary Table 4A: Transcription factor targets of the genes that increase upon *in vitro* steroids treatment of CD4 T-cells

Gene Set	Size	Expect	Ratio	P Value	FDR
V$MAX_01	260	2.2648	3.9739	0.00044	0.18195
TGACATY_UNKNOWN	661	5.7577	2.6052	0.000596	0.18195
V$PEA3_Q6	255	2.2212	3.6016	0.001726	0.30886
V$AML_Q6	264	2.2996	3.4788	0.002144	0.30886
CTGCAGY_UNKNOWN	764	6.6549	2.254	0.002528	0.30886
V$LBP1_Q6	222	1.9338	3.6199	0.003254	0.33137
V$FOXO1_02	240	2.0906	3.3484	0.004981	0.43481
V$MYCMAX_03	252	2.1951	3.1889	0.006468	0.49398
CACGTG_V$MYC_Q2	1029	8.9633	1.8966	0.007577	0.5144
V$COREBINDINGFACTOR_Q6	272	2.3693	2.9545	0.009649	0.55659

Supplementary Table 4B: Transcription factor targets of the genes that decrease upon *in vitro* steroids treatment of CD4 T-cells

Gene Set	Size	Expect	Ratio	P Value	FDR
V$CREL_01	256	1.5268	7.8596	3.31E-08	2.02E-05
V$NFKAPPAB65_01	237	1.4135	7.7822	1.44E-07	4.41E-05
GGGNNTTTCC_V$NFKB_Q6_01	134	0.79918	10.01	1.25E-06	0.000255
V$NFKB_Q6_01	232	1.3837	5.7818	6.95E-05	0.010616
TAATTA_V$CHX10_01	805	4.8011	2.916	0.000252	0.030825
V$IRF_Q6	241	1.4373	4.8701	0.000572	0.049808
V$NFKAPPAB_01	251	1.497	4.6761	0.000727	0.049808
V$NFKB_Q6	253	1.5089	4.6391	0.000762	0.049808
V$NFKB_C	263	1.5685	4.4627	0.000957	0.049808
V$AML1_01	263	1.5685	4.4627	0.000957	0.049808

Supplementary Table 4C: Transcription factor of the genes that increase upon in vitro activation of S- specific CD4 T-cells

Gene Set	Size	Expect	Ratio	P Value	FDR
TGGAAA_V$NFAT_Q4_01	1891	26.266	2.0559	7.56E-08	4.62E-05
V$COREBINDINGFACTOR_Q6	272	3.7781	4.235	1.22E-06	0.000254
TTCNRGNNNNTTC_V$HSF_Q6	151	2.0974	5.7214	1.25E-06	0.000254
V$NGFIC_01	255	3.5419	3.6703	5.65E-05	0.008341
V$AML_Q6	264	3.667	3.5452	8.05E-05	0.008341
RYTTCCTG_V$ETS2_B	1083	15.043	2.0608	8.2E-05	0.008341
V$NFKB_Q6_01	232	3.2225	3.7238	9.56E-05	0.008341
RGAANNTTC_V$HSF1_01	444	6.1672	2.7565	0.00015	0.010102
TTCYRGAA_UNKNOWN	325	4.5142	3.1013	0.000177	0.010102
V$HMGIY_Q6	248	3.4447	3.4836	0.000179	0.010102
